# Midwifery Students’ Experiences Obtaining Clinical Placements and Associations With Training Quality: “I Had to Figure It Out on My Own”

**DOI:** 10.1111/jmwh.70097

**Published:** 2026-03-23

**Authors:** Laura D. Lindberg, Julie Blumenfeld, Katherine Tierney, Amy Alspaugh

**Affiliations:** ^1^ School of Public Health, Rutgers The State University of New Jersey Newark New Jersey; ^2^ Midwifery Program, Division of Advanced Nursing Practice, School of Nursing, Rutgers The State University of New Jersey Newark New Jersey; ^3^ Department of Sociology Western Michigan University Kalamazoo Michigan; ^4^ College of Nursing University of Tennessee Knoxville Tennessee

**Keywords:** clinical education, midwifery, multimethods, precepting, survey, United States

## Abstract

**Introduction:**

Hands‐on clinical education is essential to midwifery training, yet the US system relies heavily on informal networks of clinical preceptors. Challenges in securing placements may affect students’ preparedness and satisfaction with training, but empirical data are limited. This study examines how responsibility for and difficulty in securing clinical placements are associated with the early‐career midwives’ reports of the quality of their midwifery clinical education.

**Methods:**

We conducted a national cross‐sectional survey in 2024 of certified nurse‐midwives (CNMs) and certified midwives (CMs) who received American Midwifery Certification Board certification between 2019 and 2024. The primary independent variable measured the process of finding a clinical placement, combining the responsibility for finding a placement (school vs student) with the difficulty of securing it. Logistic and linear regressions assessed associations with multiple measures of training quality, adjusting for age, race/ethnicity, degree type, year of certification, and number of placements. Open‐ended responses were analyzed thematically.

**Results:**

Fewer than half of respondents (46.0%) reported that their school had primary responsibility for their placements, 37.8% arranged their own placements with difficulty, and 16.2% arranged their own placements without difficulty. Compared with other students, those who were able to find their own clinical placements without difficulty had higher‐quality clinical education across all measured outcomes, including greater satisfaction, a greater sense of competence as a midwife, better clinical learning environments, and more positive perceptions of preceptor support and midwifery‐aligned philosophy. Qualitative findings described high stress, lack of school support, and reliance on personal networks to secure placements.

**Discussion:**

The burden of finding clinical placements negatively affects student clinical training experiences. Variability and inequity in the current clinical placement system threaten the quality of midwifery education and, ultimately, the strength of the midwifery workforce. A structured, equitable, and formalized clinical placement system is needed to ensure consistent, high‐quality midwifery education.

## INTRODUCTION

Hands‐on clinical education is an essential part of formal midwifery education and training for both certified nurse‐midwives (CNMs) and certified midwives (CMs). This vital component of midwifery education impacts students’ experience and the adequacy of their preparation to enter the workforce.^1^, ^2^ Paradoxically, the US midwifery education model depends on a largely informal network of clinical preceptors to create an appropriate learning environment—namely, one that is safe, supportive, reliable, and effective.^3^, ^4^ Despite its importance, the clinical placement process is subject to scant research and critical evaluation.

Although midwifery programs’ accreditation requires them to secure clinical placements for all students, the extent to which this is actualized is unknown.^5^ The meaning of *secure* is not operationally defined. Programs may interpret secure to mean a variety of tasks, ranging from ensuring a contract is in place with a practice or institution once a student obtains a site and preceptor themself to ensuring a student is placed with a specific preceptor at a specific site that is a seemingly good match to provide both support and education. Anecdotally, many students are left to independently find placements; students’ sometimes plaintive requests for assistance finding a clinical preceptor through social media, direct emails, American College of Nurse‐Midwives (ACNM) Connect, or other sources suggest that students face difficulties in finding the required clinical placements. In its 2024 report about midwives, the US Government Accountability Office (GAO) reports that a primary barrier faced by midwifery students is a lack of timely access to opportunities for supportive, hands‐on clinical education to meet their educational requirements.^6^ In interviews, midwifery program directors report that their lack of access to clinical sites can lead to delays in students’ progression or graduation, resulting in additional costs to students.^6^ Similarly, the ACNM Student and New Midwife Committee, representing student midwives enrolled in ACME‐accredited programs in the United States, highlights the challenge of finding adequate clinical placement opportunities.^7^

These challenges in finding clinical placements may ultimately influence the quality of clinical training because not all students report supportive clinical education experiences. In their 2022 annual report to the ACNM, students and new midwives described the negative aspects of clinical learning, including preceptors’ racism, microaggressions and disrespect, and a disparaging and judgmental approach when instructing.^7^ Additionally, some students reported a medical approach to care rather than a focus on the midwifery model taught in the classroom.^7^ Research to date has not identified the frequency of these challenges for students in finding adequate clinical placements or their impact on the quality of the learning experience.

**QUICK POINTS**

✦ The process of securing clinical placements for midwifery students varies widely by institution, and scant data have evaluated how the placement process impacts student outcomes.
✦ Students who did not experience difficulty securing their own placements, often through personal networks, had better learning environments, greater satisfaction, and stronger alignment with midwifery values.
✦ Those who had difficulty securing their own clinical site were less likely to express satisfaction and competency, but those whose program secured their own sites did not fare better.
✦ Findings highlight the urgent need for a structured, equitable clinical placement system to support student well‐being and workforce readiness in midwifery education.

In this research, we used a multimethod approach to investigate the process of securing clinical placements, identifying school versus student responsibility for this process, and the ease or difficulty a student experienced in securing their placement. We examine how these factors are associated with students’ assessment of the quality of and satisfaction with their clinical training. We draw on both closed‐ and open‐ended responses to a national survey of midwives licensed within the past 5 years to understand recent student experiences and to inform improvements in the clinical education experience for all student midwives.

## METHODS

### Study Design

This was a national cross‐sectional survey of CNMs and CMs in the United States conducted in 2024. The protocol was approved by the Rutgers University institutional review board. The Checklist for Reporting Results of Internet E‐Surveys is available (Supporting Information: Appendix S1).

### Participants

The study population was CNMs and CMs who received their certification from the American Midwifery Certification Board (AMCB) from 2019 to 2024 (n = 4117). All CNMs and CMs are required to hold certification from the AMCB, and approximately 80% of these individuals have opted in to receive research requests; the research protocol was approved by AMCB and conducted in accorded with their policies. After providing consent, respondents’ reported their date of certification to ensure the inclusion criterion was followed. We received a total of 687 surveys; 47 were excluded because of fewer than half of the survey items being completed (most of these respondents answered only a few initial survey items), resulting in 640 completed surveys. The response rate for complete surveys was 16%.

### Recruitment

Potential respondents received up to 4 invitations via email with a link to the survey from August to November of 2024. Respondents were offered a $10 electronic gift card for participation; this was increased to $30 in the fourth recruitment email to increase response rates. Additionally, all participants could choose to be entered in a raffle for one of three $100 gift cards or an iPad. Study data and consent were collected and managed using Qualtrics hosted at Rutgers University.

### Measures

The survey consisted of 46 questions; 22 questions asked about clinical and precepting experiences as a student, 13 questions asked about the current work environment, and 11 questions asked about demographics. Questions included both multiple‐choice and open‐ended items. Question items were drawn from existing validated scales as well as prior related studies; some items were developed based on content expertise and the lived experience of the authors as midwifery educators. Respondents were able to review and change their answers through a back button in Qualtrics.

Our key independent variable, *process of clinical placement*, combined 2 sets of measures—respondents’ reports of who was officially responsible for finding the clinical preceptor (school or program primarily, student) and, if the student was responsible, their difficulty in finding the needed clinical preceptor placements. The survey did not provide formal definitions of this measure. We created a 3‐category variable: school primarily responsible, student responsible/difficulty finding placement, and student responsible/no difficulty finding placement.

For outcome, our quantitative analysis focused on measures of the quality of the clinical preceptorship. We created 2 dichotomous measures. The first, *competent midwife*, was whether the respondent strongly agreed with the statement, “*My preceptor/preceptors provided me the experiences I needed to be a competent new midwife*.” A second measure, *satisfaction with preceptorship*, indicated whether the respondent reported being very satisfied with their overall clinical preceptorship experience.

Additionally, we used component scales from the Midwifery Students Evaluation of Practice (MidSTEP) tool as additional outcome variables. The MidSTEP tool is a recently developed standardized measure of midwifery students’ perceptions of their clinical learning experiences that has been found to be reliable and valid in other country contexts.^8^, ^9^, ^10^, ^11^ To our knowledge, this is the first use of the MidSTEP tool with a sample of US midwives. The reliability of the MidSTEP tool within a sample of US midwives is unknown; however, in studies of Australian,^8^ Turkish,^10^ and Italian^11^ midwifery students, Cronbach's α exceeded .85.

For this survey, we used the 7‐item *clinical skill development* subscale (assessing students’ perceptions of how well their clinical placement environment supported development of their midwifery skills), a 5‐item scale of the *preceptor skill development* (assessing students’ perceptions of how their preceptor contributed to the development of their midwifery skills), and a 5‐item scale of the *preceptor philosophy of midwifery* (assessing students’ perceptions of how well the preceptors’ actions reflect a midwifery‐specific philosophy of care) from the MidSTEP tool (each item is shown in Table 3). Each scale item used a 4‐point response scale (strongly disagree, disagree, agree, strongly agree); scores could range from a minimum of 1 to a maximum of 4, with high scores indicating more positive responses. Respondents were asked to respond to these items based on their primary preceptor at their primary clinical education placement.

As far as demographics and education variables, these included self‐reported measures of midwifery degree (master's, doctor of nursing practice/other), number of clinical placements (1, 2, 3, or more), age (24‐34 years, 35‐44 years, 45‐54 years, 55‐65 years), and year of certification (single years 2019‐2024). Respondents were asked to identify their race or ethnicity as Hispanic, non‐Hispanic Black, non‐Hispanic White, or other; we created a dichotomous measure of non‐Hispanic White versus all other because of the relatively small cell counts.

### Data Analysis

We first conducted descriptive analyses of the sample. We then estimated bivariable and multivariable associations between each outcome variable and the composite measure of responsibility and difficulty finding a preceptor. Logistic regression was used for the dichotomous outcomes (competent midwife, satisfaction), and linear regression was used for the scale variables (clinical skill development, preceptors’ skills development, philosophy of midwifery). All respondent demographic characteristics examined in the bivariable models were included as covariates in the multivariable models, given their theoretical relevance to understanding the quality of clinical preceptorship. Respondents with missing data on any of the measures in the multivariable models were excluded from the full analysis, resulting in an analytic sample of 598 respondents.

Open‐ended comments were coded using the steps of thematic analysis: familiarization with the data, generating initial codes, collating codes into themes, reviewing and refining themes, defining and naming themes, and synthesizing the findings.^12^ We used the web version of Atlas.ti for data management, coding, and analysis. For this research question, we coded participant responses that pertained to the process of finding a clinical placement only. Each response was individually coded by either the last author or student coders, and then each code was reviewed by another member of the coding team. Discrepancies and differences were reviewed in team coding meetings, and the entire team came to a consensus on the final organization of themes related to this research question.^13^

### Positionality

The second and fourth authors are CNMs. Their experiences in their midwifery clinical education influenced their understanding of this topic and their engagement with this study. The second author is a midwifery program director, who has deep knowledge of the placement process from the perspective of how her program functions. The first and third authors are both quantitative sociologists studying women's health care with extensive survey design and analysis experience. Throughout the study, we reflected on how our subjective experiences, social identities, and positionalities might influence codes, their interpretations, and the underlying assumptions that informed them.

## RESULTS

### Participant Characteristics

Participant characteristics are summarized in Table 1. More respondents received their certification in 2024 (22.6%) than in any individual year from 2019 to 2023. More than 4 out of 5 (82.1%) respondents received a master's degree. Slightly fewer than half (43.6%) of respondents were aged younger than 35 years at the time of the survey, 37.8% were aged between 35 and 45 years, and the remainder were aged more than 45 years. Three‐quarters (74.1%) identified as non‐Hispanic White. Approximately 1 in 10 respondents identified as Hispanic (8.4%) or non‐Hispanic Black (11.2%), and 6.4% identified as multiple or other races.

**Table 1:** Analytic Sample Description, SIMPLE Survey (N = 598)

Characteristic	n (%)
**Year Certified**	
2019	84 (14.0)
2020	84 (14.0)
2021	96 (16.1)
2022	104 (17.4)
2023	95 (15.9)
2024	135 (22.6)
**Degree**	
Master's	491 (82.1)
DNP/Other	107 (17.9)
**Age, y**	
<35	261 (43.6)
35‐45	226 (37.8)
>45	111 (18.6)
**Race/Ethnicity (Dichotomized)**	
Hispanic, Black, or other/multiple	155 (25.9)
White, non‐Hispanic	443 (74.1)
**Race/Ethnicity**	
Hispanic	50 (8.4)
Black, non‐Hispanic	67 (11.2)
White, non‐Hispanic	443 (74.1)
Other/multiple, non‐Hispanic	38 (6.4)

Abbreviations: DNP, doctor of nursing practice; SIMPLE, Strategies to Improve Midwifery Preceptorship and Learning Experiences.

### Clinical Training Experiences

Fewer than half of respondents (46.0%) reported that their school or program had primary responsibility for finding their clinical placements (Table 2). More than one‐third (37.8%) of respondents reported having either primary or shared responsibility and described it as difficult to find a placement, whereas the remaining 16.2% had primary or shared responsibility but did not find it difficult to find a placement. Many respondents (57.4%) reported having 3 or more different clinical training placements, 30.3% had 2 placements, and 12.4% had a single placement.

**Table 2:** Experience With and Quality of Clinical Placement (N = 598)

Characteristic	n (%)
**Process of Finding Clinical Placement**	
School responsible	275 (46.0)
Student responsible, difficult	226 (54.0)
Student responsible, not difficult	97 (16.2)
**Number of Clinical Placements**	
One	74 (12.4)
Two	181 (30.3)
Three	343 (57.4)
**Quality of Clinical Preceptorship**	
Competent midwife	372 (62.2)
Satisfied with preceptorship	344 (57.5)

Respondents reported generally positive preceptorship experiences. Nearly two‐thirds (62.2%) strongly agreed that their preceptorship helped them become a competent midwife. More than half of respondents (57.5%) reported being very satisfied with their overall preceptorship experience.

Mean values on the 3 MidSTEP scales ranged narrowly from 3.3 to 3.6 (on a 4‐point Likert scale), reflecting generally high levels of agreement with the component items (Table 3). Individual items in the clinical skill development scale with markedly lower scores were that the placement “allowed for practicing self‐care” (mean, 3.03) and “provided opportunities to voice concerns” (mean, 3.09). Individual items in the philosophy of midwifery practice scale with markedly lower scores were “preceptor role‐modeled self‐care” (mean, 3.12) and “preceptor created a sense of belonging” (mean, 3.18). In contrast, the mean scores for items in the preceptor skill development scale narrowly ranged from 3.5 to 3.7. To validate the scales within this US population, we calculated the Cronbach's α and intraclass correlation coefficient (ICC) for each scale. In this sample, the subscale Cronbach's α scores were .84 for clinical skill development, .91 for preceptor skill development, and .89 for philosophy of midwifery practice, with ICCs of 0.84, 0.94, and 0.94, respectively.

**Table 3:** MidSTEP Subscales

	Strongly Disagree %	Somewhat Disagree %	Somewhat Agree %	Strongly Agree %	Mean (SD)	Cronbach's α (95% CI)	ICC (95% CI)
**Clinical Skill Development** **Placement environment**						.836 (0.806‐0.862)	0.836 (0.816‐0.856)
supported professional growth	2	3	24	71	3.63 (0.66)		
enabled full scope of practice	3	10	31	56	3.40 (0.79)		
provided opportunities to meet requirements	2	6	19	72	3.62 (0.69)		
allowed for practicing self‐care	10	17	34	39	3.03 (0.98)		
provided self‐directed approach to learning	2	8	42	48	3.35 (0.72)		
provided opportunities to voice concerns	8	17	31	43	3.09 (0.96)		
Placement staff understood requirements	3	9	29	60	3.46 (0.76)		
**Preceptors’ Skill Development** **Preceptor**						.914 (0.892‐0.930)	0.914 (0.902‐0.924)
supported skill development	3	3	21	73	3.65 (0.67)		
understood degree requirement	2	5	23	70	3.61 (0.68)		
fostered confidence	6	6	20	69	3.52 (0.83)		
supported me in achieving clinical requirement	2	3	17	78	3.71 (0.62)		
supported me in performing clinical skills	2	4	19	75	3.67 (0.65)		
**Philosophy of Midwifery** **Preceptor**						.893 (0.874‐0.910)	0.893 (0. 879‐0.906)
role‐modeled self‐care	9	16	29	46	3.12 (0.98)		
created sense of belonging	8	15	28	49	3.18 (0.97)		
created opportunities for sharing best practice	3	8	29	60	3.46 (0.77)		
valued my clinical opinion	6	8	29	57	3.37 (0.87)		
supported me in advocating for patients’ rights	4	5	28	64	3.51 (0.76)		

Abbreviations: ICC, intraclass correlation coefficient; MidSTEP, Midwifery Student Evaluation of Practice.

### Association Between Finding a Preceptor and Outcomes

Table 4 presents bivariable and multivariable models testing the association between the responsibility for and difficulty of finding a preceptor and multiple training outcomes (sense of competence, satisfaction with training, clinical learning environment, preceptor skill development, philosophy of midwifery practice). The pattern of findings is strongly consistent. In nearly every regression, students who were responsible for finding their own placement but did not report difficulty were significantly more likely to report positive outcomes than other students. For instance, students who had responsibility without difficulty were 1.91 times as likely in the adjusted model to report being very satisfied with their preceptorship experience as students with responsibility with difficulty. These students also reported modestly higher scores on the MidSTEP scales, with effect sizes ranging from 0.16 to 0.20 in the adjusted linear models. Additionally, respondents who found their own placement without difficulty were significantly more likely to report positive outcomes for each measure examined than when the school had primary responsibility. In contrast, no significant differences were estimated between respondents whose schools were responsible for placements and those who had responsibility and experienced difficulty.

The demographic and program variables—age, race and ethnicity, program type, and year of graduation—were generally not significantly associated with the outcome measures, whereas the number of placements showed mixed associations (not shown; full model estimates are available in Supporting Information: Appendices 1 and 2).

**Table 4:** Unadjusted and Adjusted Regression Results^a^

Outcome		Student responsible, Difficult	School responsible	*P* Value^b^	Student Responsible, Not Difficult	*P* Value^b^	*P* Value^c^
Strongly agree preceptor helped me to become a competent midwife^d^	OR	1 [Reference]	0.90	.555	1.71	.045	.013
			(0.63 to 1.29)		(1.01 to 2.88)		
	AOR	1 [Reference]	0.86	.456	1.45	.186	.074
			(0.57 to 1.29)		(0.84 to 2.50)		
Very satisfied with preceptorship experience^d^	OR	1 [Reference]	1.12	.516	2.29	.002	.006
			(0.79 to 1.60)		(1.37 to 3.83)		
	AOR	1 [Reference]	1.29	.208	1.91	.018	.17
			(0.87 to 1.92)		(1.12 to 3.26)		
Clinical skill development scale	Unadj β	1 [Reference]	−.03	.525	.23	.001	>.001
			(−0.13 to 0.07)		(0.10 to 0.36)		
	Adj β	1 [Reference]	−.03	.542	.19	.008	.003
			(−0.14 to 0.08)		(0.05 to 0.33)		
Preceptor skill development scale	Unadj β	1 [Reference]	0.05	.368	.19	.009	.043
			(−0.06 to 0.15)		(0.05 to 0.33)		
	Adj β	1 [Reference]	.03	.664	.16	.038	.100
			(−0.09 to 0.14)		(0.01 to 0.30)		
Philosophy of midwifery practice scale	Unadj β	1 [Reference]	.09	.175	.27	.003	.041
			(−0.04 to 0.22)		(0.09 to 0.44)		
	Adj β	1 [Reference]	.10	.157	.20	.029	.309
			(−0.04 to 0.24)		(0.02 to 0.38)		

Abbreviations: Adj, adjusted; aOR, adjusted OR; OR, odds ratio; Unadj, unadjusted.

95% CIs in parentheses.

a All adjusted models control for age, race and ethnicity, program type, year certified, and number of placements.

b For comparisons when *student responsible, difficult* is the reference group.

c For comparisons when *school responsible* is the reference group.

d Logistic regression with exponentiated coefficients presented. All other models used ordinary least squares regression.

### Open‐Ended Responses That Illuminate Quantitative Findings

Analysis of open‐ended questions provided more nuance to what contributed to their difficult or easy time finding a clinical placement. Major themes identified during data analysis included: having a hard time finding placement, lack of assistance from one's program, the stress and anxiety of finding a clinical placement, and the commonality of precepting within known networks.

Most notable were those who shared the ramifications of having a hard time finding placements. One person who was primarily responsible for finding their placement and had a very difficult time said that the worst part of her clinical education was:

The fact that I had to beg people to try and find intrapartum clinical sites. The only reason I was able to do it at my work was because I had a great boss who I told I was going to fail if I couldn't find a placement to complete my intrapartum clinical rotation. She let me take 2 months of leave, while the midwives that I had a great relationship with precepted me. (#247)

Another shared:

I lost clinical placement during the start of the pandemic and had to fight very hard to regain placement. So many practices have already promised time to students. Many practices have obligations to other types of students. Midwives are not prioritized. This was a very negative experience that had a deep impact on my mental health. I almost left my program multiple times. (#504)

Additionally, many in our study reported little or no assistance from their school. One participant, who reported having a very difficult time finding a clinical placement, summed this experience up: “The school was no help, and I had to figure it out on my own” (#496). Another participant responded that the worst part about their clinical education was, “Not having adequate support from my school to find and get my preceptorships” (#36). Some midwives reported receiving assistance in finding a clinical placement, but it often fell short of being truly helpful. One explained:

My entire clinical placement was such a mess. It took a long time to secure placement. Everything was pieced together. Very stressful. My school did not help ‐ they provided me a list online of people who had precepted in the past. Most of the providers I worked with were burnt out and exhausted. (#375)

Numerous individuals noted that they were given out‐of‐date resources that did not aid in their search for clinical sites.

Furthermore, participants who had a difficult time finding their placements reported high levels of stress and anxiety during this process. One participant described the process of finding clinical placements:

It was like a full‐time job finding placement. I moved my family 2,000 miles to accommodate my training, and then my placements fell through, and I had to scramble to start over. I found a new placement, and she decided we weren't a good fit before I even started working with her. I had to scramble again. The stress from that affected not only me but my family, and we still sometimes process this struggle. (#330)

Placements that fell through were more often mentioned by participants who had to find their own clinical placement sites. Another participant shared the impact of this stress, saying, “I almost quit midwifery school with 2 semesters remaining because I could not find preceptors, it was so stressful when I had already invested so much time and money” (#520).

The open‐ended responses also helped identify how common it is for students to precept within known networks. Typically, those who knew their preceptors or were familiar with their clinical site previously were more likely to report that their placement was easy to find. Sometimes, participants reported that they had worked as a labor and birthing nurse at their site before their midwifery education, as explained by one participant: “I was already familiar with all the providers as I worked as a labor and delivery nurse at this facility” (#601). Those who were familiar with their clinical site tended to have a more positive experience with their clinical education. One participant who had a somewhat easy time finding a placement reported, “My primary [site] was phenomenal. Local area and practice that I knew and worked at. The flow was familiar to me” (#629). Learning in a known environment has certain other benefits, as explained by one participant: “I knew my preceptors and all the nurses I worked with. I was already respected” (#638). Another benefit of using a known network of colleagues was decreased stress, which was exemplified by one participant who said that the best part of their clinical education was “[t]he ease of finding a preceptor and clinical site because of personal connections and the site already being approved from previous students” (#685).

## DISCUSSION

This national survey of new CNMs and CMs reveals that the process of securing clinical placements has significant implications. The association between the experience of finding a clinical preceptor and the measured outcomes is remarkably consistent. Respondents who had some responsibility for arranging their own placements and reported difficulty doing so reported worse experiences across all measured outcomes compared with those who did not struggle to find their placements. In contrast, outcomes were similarly poor between respondents whose schools were responsible for placements and those who had responsibility and experienced difficulty. This suggests that schools may also struggle to secure high‐quality, appropriate sites and highlights the need for a structured, formalized, and sustained system to intentionally match students with effective clinical sites. Those who were able to find their own preceptorships without difficulty had better learning outcomes than other students. In this case, students “matched” on their own, with positive outcomes. However, students with difficulty finding their own match or relying on their school did not fare as well.

In addition to general measures of satisfaction and competence, the MidSTEP scales offered a structured lens through which to assess the quality of clinical education. Our study is the first to apply the scale with a US sample of midwives, and the high‐reliability measures indicate its fit within this context. The scales systematically capture specific dimensions of clinical education, including the learning environment, skill development, and alignment with the midwifery model of care. Through this tool, we identified consistent and compelling patterns: Respondents who faced difficulties in securing placements scored lower across all MidSTEP domains, indicating reduced opportunities for clinical growth, mentorship, and a sense of integration into the midwifery community. In contrast, students with easier access to placements scored significantly higher, highlighting how well‐matched placements can foster clinical competence, professional confidence, and a strong sense of belonging. Future research should consider including the MidSTEP scales in other surveys and continue to explore factors that promote or impede more positive evaluations.

Together, the findings of this study provide both quantitative and qualitative support for earlier reports by the US GAO and the ACNM Student and New Midwife Committee, which highlighted the lack of access to timely, high‐quality clinical experiences as a persistent barrier for midwifery students.^6^, ^7^ Our results add nuance by showing that how placements are arranged, and the perceived difficulty of that process, shapes not only students’ logistical success in meeting program requirements but also their deeper sense of professional readiness and well‐being.

These findings are not unique to the profession of midwifery. Within advanced practice nursing education in the United States and Canada, similar challenges exist; there is no formal system in place to secure clinical preceptors or sites.^14^, ^15^ This leads to similar challenges in the ability to secure and maintain clinical site placements for these students. Conversely, in the United States, a robust, structured system exists for obstetric resident placements, perpetuating the strength of this workforce.^16^

Our findings also highlight the need to address clinical placements across the field of midwifery. Interpretation of the AMCB accreditation requirement that academic programs “secure” clinical placements for their students varies widely across academic institutions; more than half of the respondents had some responsibility for arranging their own placements, and most of them reported difficulty doing so. This often arduous process has significant ramifications for students. Furthermore, different schools may have diverse challenges in securing preceptors for their students, depending on factors such as geographic location, financial resources, and the ability to draw on an established network of alumni or other preceptors.^3^ Despite such obstacles, relying on students is not a useful or acceptable alternative. In light of our findings that students often feel immense pressure and stress when having a hard time securing their placements, the profession must advocate for and actualize a structured, sustained system to support midwifery programs to ensure readily accessible, high‐quality clinical education.

### Implications for Policy and Practice

Our study underscores the need to systematize and strengthen the clinical education infrastructure in US midwifery training. Based on our findings, we recommend considering the following policy and practice changes.

One approach would be to undertake a feasibility study examining the creation of a national, formalized system for clinical placement matching in midwifery education modeled after systems used in other health care professions.^17^ Although such a system could offer clear benefits, including reducing the administrative burden on individual education institutions, improving equity in placement access, and enhancing the overall quality of clinical experiences, the logistical and financial challenges are substantial. Developing and sustaining a centralized or regionally coordinated matching platform would require significant coordination among education programs, preceptors, and health care systems. Given existing institutional barriers to precepting,^18^ exploring this possibility could be a critical step toward addressing long‐standing inequities and inefficiencies in midwifery clinical education.

An additional strategy to mitigate current obstacles to the clinical site placement process is to further expand and incentivize preceptorship. Although currently preceptors may earn continuing education credits for precepting, additional incentives may broaden the pool of interested midwives. Other stakeholders, such as state governments, health care systems, and insurers who benefit from the growth of the midwifery workforce, should provide stronger support and incentives to midwives to serve as preceptors. Our prior work indicates that the inclusion of precepting in job descriptions, financial stipends, and workload relief may increase participation.^19^ Another possibility includes placing 2 students with one preceptor, thereby decreasing the strain on an already small workforce.^20^

This study has several relevant strengths and limitations. It uses survey data from a recent large and nationally representative sample of new CMs; the respondents are a slightly more diverse group than the most recent national workforce data.^21^ The survey used valid and reliable survey tools, incorporating both quantitative and qualitative data that help broaden our understanding of clinical education. However, students who did not complete education programs because of a lack of clinical site placement or negative experiences with the same were not included in the pool of respondents. Although the response rate is consistent with similar surveys of health care professionals, there is still concern about the potential for unknown bias because of differential nonresponse and incomplete survey data, which may affect generalizability and could not be directly assessed.

Finally, this work is from the perspective of new midwifery students and does not represent the experiences and perspectives of those who place or precept midwifery students. Subsequent research needs to explore characteristics of the educational setting, including whether they have existing and robust faculty practices that may mitigate the challenges of finding clinical education sites. Other future research directions include exploring faculty practice models, researching the development of non–school‐based placement systems, and studying longer‐term outcomes, such as burnout and career satisfaction.

## CONCLUSION

A midwifery student's journey to clinical competence should not depend on luck, privilege, or personal connections. The variability and inequity in the current clinical placement system threaten the quality of midwifery education and, ultimately, the strength of the midwifery workforce. Our findings call for urgent investment in a more structured, supportive, and equitable system for clinical education that aligns with the values of midwifery and upholds a collective commitment to preparing future midwives for safe, respectful, and holistic care.

## CONFLICT OF INTEREST 

The authors have no conflicts of interest to disclose.

## Supporting information




**Appendix S1**. Full Adjusted Regression Models with Student Responsible, Difficult as Reference Group (n = 598).

**Appendix S2**. Consolidated criteria for Reporting Qualitative research Checklist (COREQ)

**Appendix S3**. Checklist for Reporting Results of Internet E‐Surveys (CHERRIES).
