# Supplementary material for: Midwifery Students’ Experiences Obtaining Clinical Placements and Associations With Training Quality: “I Had to Figure It Out on My Own”
Source: J Midwifery Womens Health. 2026 Mar 23;71(3):395–403. doi: 10.1111/jmwh.70097 (PMC13263908; doi:10.1111/jmwh.70097)
Supplement: Supplementary file 1 — Appendix S1. Full Adjusted Regression Models with Student Responsible, Difficult as Reference Group (n = 598). [file JMWH-71-395-s001.docx]

**Appendix 1**: Full Adjusted Regression Models with Student Responsible, Difficult as Reference Group (n=598)

|  | **Strongly Agree Preceptor Helped me to Become a Competent Midwife (a)** |  | **Very Satisfied with Preceptor-ship Experience (a)** |  | **Clinical Skill Development Scale** | |  | **Preceptor Skill Development Scale** | |  | **Philosophy of Midwifery Practice Scale** | |  |
| --- | --- | --- | --- | --- | --- | --- | --- | --- | --- | --- | --- | --- | --- |
|  | *AOR* | *P* Value | *AOR* | *P* Value | *Adj B* | | *P* Value | *Adj B* | | *P* Value | *Adj B* | | *P* Value |
| **Placement (Ref = Student Responsible, Difficult)** |  |  |  |  |  | |  |  | |  |  | |  |
| School responsible | 0.86 | 0.456 | 1.29 | 0.208 | -0.03 | | 0.542 | 0.03 | | 0.664 | 0.10 | | 0.157 |
|  | (0.57-1.29) |  | (0.87-1.92) |  | (-0.14-0.08) | |  | (-0.09-0.14) | |  | (-0.04-0.24) | |  |
| Student Responsible, Not Difficult | 1.45 | 0.186 | 1.91 | 0.018 | 0.19 | | 0.008 | 0.16 | | 0.038 | 0.20 | | 0.029 |
|  | (0.84-2.50) |  | (1.12-3.26) |  | (0.05-0.33) | |  | (0.01-0.30) | |  | (0.02-0.38) | |  |
| **Age Group (Ref <35)** |  |  |  |  |  | |  |  | |  |  | |  |
| 35-44 | 0.65 | 0.034 | 0.89 | 0.545 | -0.05 | | 0.378 | -0.10 | | 0.074 | -0.10 | | 0.164 |
|  | (0.44-0.97) |  | (0.60-1.31) |  | (-0.15-0.06) | |  | (-0.21-0.01) | |  | (-0.23-0.04) | |  |
| >45 | 0.73 | 0.208 | 1.28 | 0.321 | -0.05 | | 0.468 | -0.02 | | 0.777 | 0.03 | | 0.700 |
|  | (0.44-1.19) |  | (0.78-2.09) |  | (-0.18-0.08) | |  | (-0.16-0.12) | |  | (-0.14-0.20) | |  |
| **Race/Ethnicity (Ref = NH white)** |  |  |  |  |  | |  |  | |  |  | |  |
| NH non-white | 1.28 | 0.237 | 0.86 | 0.455 | -0.00 | | 0.951 | 0.02 | | 0.759 | 0.05 | | 0.474 |
|  | (0.85-1.91) |  | (0.59-1.27) |  | (-0.11-0.10) | |  | (-0.09-0.13) | |  | (-0.09-0.19) | |  |
| **Program Type (Ref = Masters)** |  |  |  |  |  | |  |  | |  |  | |  |
| DNP/Other | 0.78 | 0.288 | 1.10 | 0.686 | 0.02 | | 0.738 | 0.05 | | 0.445 | 0.01 | | 0.879 |
|  | (0.50-1.23) |  | (0.70-1.72) |  | (-0.10-0.14) | |  | (-0.08-0.18) | |  | (-0.15-0.17) | |  |
| **Year Certified (Ref = 2019)** |  |  |  |  |  | |  |  | |  |  | |  |
| 2020 | 2.35 | 0.010 | 0.77 | 0.420 | -0.03 | | 0.686 | 0.01 | | 0.891 | -0.07 | | 0.549 |
|  | (1.23-4.50) |  | (0.41-1.45) |  | (-0.20-0.13) | |  | (-0.17-0.19) | |  | (-0.29-0.15) | |  |
| 2021 | 1.33 | 0.354 | 0.67 | 0.196 | -0.02 | | 0.847 | -0.01 | | 0.910 | 0.09 | | 0.384 |
|  | (0.73-2.44) |  | (0.36-1.23) |  | (-0.18-0.15) | |  | (-0.19-0.17) | |  | (-0.12-0.31) | |  |
| 2022 | 1.18 | 0.576 | 0.64 | 0.151 | 0.02 | | 0.837 | -0.06 | | 0.461 | 0.01 | | 0.904 |
|  | (0.66-2.14) |  | (0.35-1.17) |  | (-0.15-0.18) | |  | (-0.24-0.11) | |  | (-0.20-0.22) | |  |
| 2023 | 1.80 | 0.066 | 0.77 | 0.400 | 0.04 | | 0.647 | 0.11 | | 0.214 | 0.18 | | 0.110 |
|  | (0.96-3.37) |  | (0.41-1.43) |  | (-0.13-0.21) | |  | (-0.07-0.29) | |  | (-0.04-0.39) | |  |
| 2024 | 1.61 | 0.106 | 0.99 | 0.975 | 0.17 | | 0.035 | 0.04 | | 0.625 | 0.18 | | 0.084 |
|  | (0.90-2.88) |  | (0.55-1.78) |  | (0.01-0.33) | |  | (-0.13-0.21) | |  | (-0.02-0.38) | |  |
| **Placements (Ref = One)** |  |  |  |  |  | |  |  | |  |  | |  |
| Two | 0.55 | 0.066 | 0.41 | 0.006 | -0.20 | | 0.015 | -0.09 | | 0.303 | -0.27 | | 0.009 |
|  | (0.29-1.04) |  | (0.22-0.77) |  | (-0.35--0.04) | |  | (-0.26-0.08) | |  | (-0.47--0.07) | |  |
| Three or more | 0.54 | 0.055 | 0.37 | 0.002 | -0.18 | | 0.020 | -0.09 | | 0.287 | -0.28 | | 0.006 |
|  | (0.29-1.01) |  | (0.20-0.70) |  | (-0.34--0.03) | |  | (-0.25-0.07) | |  | (-0.48--0.08) | |  |
| **Constant** | 2.42 | 0.026 | 3.28 | 0.003 | 3.50 | >0.001 | | 3.68 | >0.001 | | 3.43 | >0.001 | |
|  | (1.11-5.28) |  | (1.50-7.17) |  | (3.30-3.70) | |  | (3.47-3.90) | |  | (3.18-3.69) | |  |

95% Confidence Intervals in Parentheses.

Notes:

1. Logistic regression with exponentiated coefficients presented. All other models utilized OLS regression.
2. Abbreviations: OR = Odds-Ratio; aOR = Adjusted Odds-Ratio; Unadj = Unadjusted; Adj = Adjusted ; NH = non-Hispanic ; DNP = Doctor of Nursing

**Appendix 2**: Full Adjusted Regression Models with School Responsible as Reference Group (n=598)

|  | **Strongly Agree Preceptor Helped me to Become a Competent Midwife (a)** |  | **Very Satisfied with Preceptor-ship Experience (a)** | |  | **Clinical Skill Development Scale** | |  | **Preceptor Skill Development Scale** |  | **Philosophy of Midwifery Practice Scale** | |  |
| --- | --- | --- | --- | --- | --- | --- | --- | --- | --- | --- | --- | --- | --- |
|  | *AOR*  *(95% CI)* | *P* Value | *AOR*  *(95% CI)* | | *P* Value | *Adjusted* | | *P* Value | *Adjusted* | *P* Value | *Adjusted* | | *P* Value |
| **Placement (Ref = School Responsible)** |  |  |  | |  |  | |  |  |  |  | |  |
| Student Responsible, Difficult | 1.17 | 0.456 | 0.77 | | 0.208 | 0.03 | | 0.542 | -0.03 | 0.664 | -0.10 | | 0.157 |
|  | (0.78-1.75) |  | (0.52-1.15) | |  | (-0.08-0.14) | |  | (-0.14-0.09) |  | (-0.24-0.04) | |  |
| Student Responsible, Not Difficult | 1.69 | 0.074 | 1.48 | | 0.170 | 0.22 | | 0.003 | 0.13 | 0.100 | 0.10 | | 0.309 |
|  | (0.95-2.99) |  | (0.85-2.58) | |  | (0.08-0.37) | |  | (-0.02-0.29) |  | (-0.09-0.28) | |  |
| **Age Group (Ref <35)** |  |  |  | |  |  | |  |  |  |  | |  |
| 35-44 | 0.65 | 0.034 | 0.89 | | 0.545 | -0.05 | | 0.378 | -0.10 | 0.074 | -0.10 | | 0.164 |
|  | (0.44-0.97) |  | (0.60-1.31) | |  | (-0.15-0.06) | |  | (-0.21-0.01) |  | (-0.23-0.04) | |  |
| >45 | 0.73 | 0.208 | 1.28 | | 0.321 | -0.05 | | 0.468 | -0.02 | 0.777 | 0.03 | | 0.700 |
|  | (0.44-1.19) |  | (0.78-2.09) | |  | (-0.18-0.08) | |  | (-0.16-0.12) |  | (-0.14-0.20) | |  |
| **Race/Ethnicity (Ref = NH white)** |  |  |  | |  |  | |  |  |  |  | |  |
| NH non-white | 1.28 | 0.237 | 0.86 | | 0.455 | -0.00 | | 0.951 | 0.02 | 0.759 | 0.05 | | 0.474 |
|  | (0.85-1.91) |  | (0.59-1.27) | |  | (-0.11-0.10) | |  | (-0.09-0.13) |  | (-0.09-0.19) | |  |
| **Program Type (Ref = Masters)** |  |  |  | |  |  | |  |  |  |  | |  |
| DNP/Other | 0.78 | 0.288 | 1.10 | | 0.686 | 0.02 | | 0.738 | 0.05 | 0.445 | 0.01 | | 0.879 |
|  | (0.50-1.23) |  | (0.70-1.72) | |  | (-0.10-0.14) | |  | (-0.08-0.18) |  | (-0.15-0.17) | |  |
| **Year Certified (Ref = 2019)** |  |  |  | |  |  | |  |  |  |  | |  |
| 2020 | 2.35 | 0.010 | 0.77 | | 0.420 | -0.03 | | 0.686 | 0.01 | 0.891 | -0.07 | | 0.549 |
|  | (1.23-4.50) |  | (0.41-1.45) | |  | (-0.20-0.13) | |  | (-0.17-0.19) |  | (-0.29-0.15) | |  |
| 2021 | 1.33 | 0.354 | 0.67 | | 0.196 | -0.02 | | 0.847 | -0.01 | 0.910 | 0.09 | | 0.384 |
|  | (0.73-2.44) |  | (0.36-1.23) | |  | (-0.18-0.15) | |  | (-0.19-0.17) |  | (-0.12-0.31) | |  |
| 2022 | 1.18 | 0.576 | 0.64 | | 0.151 | 0.02 | | 0.837 | -0.06 | 0.461 | 0.01 | | 0.904 |
|  | (0.66-2.14) |  | (0.35-1.17) | |  | (-0.15-0.18) | |  | (-0.24-0.11) |  | (-0.20-0.22) | |  |
| 2023 | 1.80 | 0.066 | 0.77 | | 0.400 | 0.04 | | 0.647 | 0.11 | 0.214 | 0.18 | | 0.110 |
|  | (0.96-3.37) |  | (0.41-1.43) | |  | (-0.13-0.21) | |  | (-0.07-0.29) |  | (-0.04-0.39) | |  |
| 2024 | 1.61 | 0.106 | 0.99 | | 0.975 | 0.17 | | 0.035 | 0.04 | 0.625 | 0.18 | | 0.084 |
|  | (0.90-2.88) |  | (0.55-1.78) | |  | (0.01-0.33) | |  | (-0.13-0.21) |  | (-0.02-0.38) | |  |
| **Placements (Ref = One)** |  |  |  | |  |  | |  |  |  |  | |  |
| Two | 0.55 | 0.066 | 0.41 | | 0.006 | -0.20 | | 0.015 | -0.09 | 0.303 | -0.27 | | 0.009 |
|  | (0.29-1.04) |  | (0.22-0.77) | |  | (-0.35--0.04) | |  | (-0.26-0.08) |  | (-0.47--0.07) | |  |
| Three or more | 0.54 | 0.055 | 0.37 | | 0.002 | -0.18 | | 0.020 | -0.09 | 0.287 | -0.28 | | 0.006 |
|  | (0.29-1.01) |  | (0.20-0.70) | |  | (-0.34--0.03) | |  | (-0.25-0.07) |  | (-0.48--0.08) | |  |
| **Constant** | 2.07 | 0.075 | 4.23 | >0.001 | | 3.47 | >0.001 | | 3.71 | >0.001 | 3.53 | >0.001 | |
|  | (0.93-4.63) |  | (1.88-9.52) | |  | (3.26-3.67) | |  | (3.49-3.93) |  | (3.27-3.80) | |  |

95% Confidence Intervals in Parentheses.

Notes:

1. Logistic regression with exponentiated coefficients presented. All other models utilized OLS regression.
2. Abbreviations: OR = Odds-Ratio; aOR = Adjusted Odds-Ratio; Unadj = Unadjusted; Adj = Adjusted ; NH = non-Hispanic ; DNP = Doctor of Nursing
